# Integrated profiling of endoplasmic reticulum stress-related DERL3 in the prognostic and immune features of lung adenocarcinoma

**DOI:** 10.3389/fimmu.2022.906420

**Published:** 2022-10-07

**Authors:** Lanlan Lin, Guofu Lin, Hai Lin, Luyang Chen, Xiaohui Chen, Qinhui Lin, Yuan Xu, Yiming Zeng

**Affiliations:** ^1^ Department of Pulmonary and Critical Care Medicine, The Second Affiliated Hospital of Fujian Medical University, Quanzhou, China; ^2^ Respiratory Medicine Center of Fujian Province, Quanzhou, China; ^3^ The Second Clinical College, Fujian Medical University, Quanzhou, China; ^4^ Clinical Research Unit, The Second Affiliated Hospital of Fujian Medical University, Quanzhou, China

**Keywords:** DERL3, lung adenocarcinoma, endoplasmic reticulum stress, immune infiltration, B cell

## Abstract

**Background:**

DERL3 has been implicated as an essential element in the degradation of misfolded lumenal glycoproteins induced by endoplasmic reticulum (ER) stress. However, the correlation of DERL3 expression with the malignant phenotype of lung adenocarcinoma (LUAD) cells is unclear and remains to be elucidated. Herein, we investigated the interaction between the DERL3 and LUAD pathological process.

**Methods:**

The Cancer Genome Atlas (TCGA) database was utilized to determine the genetic alteration of DERL3 in stage I LUAD. Clinical LUAD samples including carcinoma and adjacent tissues were obtained and were further extracted to detect DERL3 mRNA expression *via* RT-qPCR. Immunohistochemistry was performed to evaluate the protein expression of DERL3 in LUAD tissues. The GEPIA and TIMER website were used to evaluate the correlation between DERL3 and immune cell infiltration. We further used the t-SNE map to visualize the distribution of DERL3 in various clusters at the single-cell level *via* TISCH database. The potential mechanisms of the biological process mediated by DERL3 in LUAD were conducted *via* KEGG and GSEA.

**Results:**

It was indicated that DERL3 was predominantly elevated in carcinoma compared with adjacent tissues in multiple kinds of tumors from the TCGA database, especially in LUAD. Immunohistochemistry validated that DERL3 was also upregulated in LUAD tissues compared with adjacent tissues from individuals. DERL3 was preliminarily found to be associated with immune infiltration *via* the TIMER database. Further, the t-SNE map revealed that DERL3 was predominantly enriched in plasma cells of the B cell population. It was demonstrated that DERL3 high-expressed patients presented significantly worse response to chemotherapy and immunotherapy. GSEA and KEGG results indicated that DERL3 was positively correlated with B cell activation and unfolded protein response (UPR).

**Conclusion:**

Our findings indicated that DERL3 might play an essential role in the endoplasmic reticulum-associated degradation (ERAD) process in LUAD. Moreover, DERL3 may act as a promising immune biomarker, which could predict the efficacy of immunotherapy in LUAD.

## Introduction

Lung cancer remains the most prevalent cause of cancer-related death worldwide (1, 2). Histologically, lung cancer is categorized into small cell lung cancer (SCLC) and non-small cell lung cancer (NSCLC) (3). Lung adenocarcinoma (LUAD) is the major subtype of NSCLC with increasing morbidity annually (4). Despite advances in the intensive investigation of molecular mechanisms underlying lung cancer and new breakthroughs in immunotherapy in NSCLC (5), the 5-year overall survival rate of lung cancer is only 11% (6). Therefore, it is necessary to elucidate the profound mechanisms and explore the potential pathogenic genetic variants of lung cancer.

DERL3 (also known as Derlin-3) is a member of the Derlin family which contains additional members, including DERL1 and DERL2 (7). DERL3 gene lies on chromosome 22q11.23 and its mature protein localizes primarily on the endoplasmic reticulum (ER) membrane (8). Previous studies indicated that DERL3 was involved in the metastasis and apoptosis of colorectal cancer (9), gastric cancer (10), and breast cancer (8). It has been reported that DERL3 was mainly involved in the endoplasmic reticulum associated degradation (ERAD) process *via* targeting SLC2A1 in the Warburg effect (11). ERAD is one of the most important mechanisms for adaptation to ER stress and intracellular quality control for eliminating unfolded and misfolded proteins (12). Collectively, the above researches indicated that aberrant expression of DERL3 might be applied as a promising biomarker in malignant tumors. Although emerging evidence has been revealed that DERL3 promoted the proliferation and migration of LUAD (13), the physiological significance and profound mechanism of DERL3 in LUAD ER stress and immune infiltration had not been elucidated.

In the present study, bioinformatics techniques were applied to comprehensively elaborate the pathway enrichment and immune infiltration of DERL3 in a tumor microenvironment (TME). Our results identified that DERL3 might be involved in the pathogenesis of lung cancer and could play an essential role in ERAD process to mediate ER stress.

## Materials and methods

### Tissue samples

Clinical samples including malignant and non-neoplastic lung tissues were obtained from The Second Affiliated Hospital of Fujian Medical University. The application of archived cancer samples was approved by the relevant Ethics Commission (approval No. 2022-89). All patients had been diagnosed as stage I LUAD based on their histological and pathological characteristics. None of the patients received any pre-operative chemotherapy or radiotherapy prior to tissue sampling. All patients were given written informed consent. Excised tissues were stored at -80°C for long-term conservation.

### Transcript data analysis

Total RNA was extracted from 10 paired stage I LUAD tissues and corresponding adjacent tissues using the RNeasy Mini Kit (Qiagen, Cat No. 74104, Germany) according to the manufacturer’s protocol. Then, rRNA was removed from the total RNA to obtain the maximum residual ncRNA. After fragmenting rRNA-depleted RNA, the cDNA library was performed using the TruSeq RNA sample Prep Kit (Illumina, RS-122-2001, USA). The mRNA sequencing libraries were prepared using the VAHTS total RNA-seq Library Prep kit for Illumina (Vazyme, NR603, China) following the manufacturer’s protocol. After sequencing, the reads files (fastq) were mapped to the Hg19 reference using STAR, and gene expression was determined using RSEM. Differential expression analysis for mRNA was performed using the DESeq2 R package (https://bioconductor.org/packages/release/bioc/html/DESeq2.html). Differentially expressed genes (DEGs) were obtained by comparing gene expressions between two groups using DESeq2 (v1.10.1). A corrected p value of <0.05 and |Log_2_ (fold change)| (|log_2_ FC|) ≥1 were considered statistically significant. Heat maps were generated using the R package with a hierarchical clustering algorithm.

### Immunohistochemistry (IHC)

Immunohistochemical staining containing 16 LUAD patients was carried out. All tissue specimens were fixed and embedded in paraffin. Antigen retrieval was performed in a pH 6.0 citrate buffer for 10 min after deparaffinization. Peroxide blocking was performed for 30 min with 3% H_2_O_2_ to block endogenous peroxidases. The slides were incubated with a primary antibody of anti-DERL3 (ab78233, abcam) at 4°C overnight, and an HRP-labeled secondary antibody for 30 min at room temperature. After peroxidase substrate DAB staining, slices were counterstained with hematoxylin for 3 min and final images were captured using a fluorescence microscope (OLYMPUS CKX41, Japan).

### Cell culture

Bronchial epithelial cell line HBE and LUAD cell lines containing A549, H1299, H1975, SPCA-1, and H460 were purchased from Cell Bank of Chinese Academy of Science (Shanghai, China). Cells were cultured in RPMI-1640 or DMEM medium (Gibco, USA) containing 10% fetal bovine serum (FBS) (Gibco, USA) and 1% penicillin–streptomycin (Beyotime, Tianjin, China) at a 37°C humidified incubator with 5% CO_2_ (Thermo Scientific, Waltham, MA, USA). The medium was replaced during incubation based on the cellular demand.

### Reverse transcription quantitative PCR (RT−qPCR)

Total RNA of 33 paired LUAD and adjacent tissues were extracted using a TRIzol^®^ reagent (Invitrogen, Thermo Fisher Scientific, Inc.) according to the manufacturer’s protocol. After the quantification and purity were confirmed, the extracted RNA was reverse-transcribed into cDNA using the Reverse Transcription Kit (Fermentas, Vilnius, Lithuania). Then, RT-qPCR was conducted on ABI 7300 Real-Time PCR system (Applied Biosystems, Foster City, CA, USA) with a SYBR Green PCR Kit (Thermo Fisher Scientific, Inc.). The primer sequences are listed in **Table S1**. The relative gene expression level was measured using the 2^-ΔΔCt^ method.

### Transfection

DERL3 small interfering RNAs (siRNAs) and DERL3-flag plasmid were synthesized by Hanheng (Shanghai, China). A549 and H1975 cells were cultured in six-well plates at 3.0 × 10^5^ cells/well overnight and transfected with siRNA using Lipofectamine 3000 (Invitrogen, Carlsbad, CA, USA) according to the manufacturer’s instructions. SPCA-1 cells were seeded into six-well plates at 4.0 × 10^5^ cells/well overnight and transfected with 1 µg of plasmid. After transfection for 48 h, cells were harvested to identify the efficiency using RT-qPCR. The sequences for DERL3 siRNA are listed in **Table S1**.

### Migration assays

Transwell migration assay was conducted to assess cellular migrative ability. The density of cells was adjusted to 5.0 × 10^4^ cells/well and resuspended in a serum-free medium; the RPMI-1640 medium containing 10% FBS was applied to the lower chambers. After incubation at 37°C for 24 h, non-invaded cells on the upper chambers were scraped out. Cells on the lower chambers were fixed in 4% paraformaldehyde and stained with 1% crystal violet (Solarbio Life Sciences, China) for 30 min. The number of cells was counted under microscope from at least three random fields.

Scratch assay was performed to assess cell migration. Cells were plated into the six-well plate with complete medium. When cells reached full confluence, scratch wounds were scraped in a straight line using a 200 μl pipette tip. Cell debris was removed using phosphate-buffered saline (PBS) and the serum-free medium was replaced. Photographs were captured at 0 h and 24 h, respectively. The scratch healing rate was calculated as follows: (scratch area of 0 h − scratch area of 24 h)/scratch area of 0 h × 100%.

### Cell Counting Kit-8 (CCK-8) assay

Cells at the density of 4.0 × 10^3^ cells/well were seeded into a 96-well plate and cultured at a 37°C incubator overnight. After incubation for 0, 24, 48, and 72 h, a 100 µl mixture of CCK-8 and serum-free medium at a volume ratio of 1:10 was added and incubated for an additional 1 h at 37°C. The absorbance value (OD) was measured at 450 nm.

### DERL3 differential expression and localization

The TIMER 2.0 database (http://timer.cistrome.org/) consists of three major components, including immune association, cancer exploration, and immune estimation. We used the “cancer exploration” module to explore DERL3 differential expression. The expression of DERL3 was further performed by Sangerbox (http://sangerbox.com/tool) using the pan-cancer monogenic fast comprehensive analysis. Furthermore, the RNA-seq dataset was obtained from The Cancer Genome Atlas (TCGA) database to analyze DERL3 expression in stage I LUAD and normal tissues.

The Human Protein Atlas (HPA) is a comprehensive resource for mapping human proteins in cells, tissues, and organs through multiple omics technologies (14). We used the “Tissue Atlas” module to present the distribution of DERL3 in pulmonary tissues.

### Kaplan–Meier survival analysis

The associations of DERL3 expression with overall survival (OS) and first progression (FP) analysis were performed using the Kaplan–Meier plotter (https://kmplot.com/analysis/). Raw counts of RNA-sequencing data and corresponding prognostic information were obtained from the TCGA dataset (https://portal.gdc.cancer.gov/) to further assess the expression of DERL3 in the different stages of lung cancer.

### UALCAN portal

UALCAN (http://ualcan.path.uab.edu) is a convenient database to provide an analysis of transcriptional expressions based on the TCGA database. We evaluated the promoter methylation level of DERL3 in normal tissues and primary pulmonary tumors based on different tumor stages.

### UCSC Xena

UCSC Xena (http://xena.ucsc.edu) empowers biologists to explore data across multiple Xena Hubs with a variety of visualizations and analyses (15). RNA-seq, phenotype profiles, and DNA methylation (Methylation 450) were accessed to validate DERL3 methylation signatures and clinicopathological features in lung cancer. The methylation level was assessed by β value. β value ≥0.6 was considered as methylated, while β value ≤0.2 was considered to be unmethylated (16). Intermediate β value was considered to be partially methylated.

### PPI network and functional enrichment analysis

Protein–protein interaction (PPI) networks were constructed using the GeneMANIA (http://www.genemania.org/). Kyoto Encyclopedia of Genes and Genomes (KEGG) and Gene Ontology (GO) functional enrichment analyses were conducted using DAVID Functional Annotation Tools (https://david.ncifcrf.gov/).

### Immune infiltration analysis

The TIMER 2.0 database was applied to investigate the relationship between immune cell infiltration and DERL3 expression. Gene Expression Profiling Interactive Analysis (GEPIA) (http://gepia.cancer-pku.cn/) is a visualization website based on TCGA datasets. The GEPIA 2021 website was further used for the correlation between DERL3 and immune cell infiltration. Immune scores and stromal scores were calculated using the ESTIMATE algorithm. The EPIC algorithm was utilized to quantify the proportions of immune cells. The infiltration abundance of immune cells in heterogeneous LUAD tissues was assessed *via* using the Microenvironment Cell Population-counter (MCP-counter) algorithm.

### Immunotherapy response and chemotherapy response

The Tumor Immune Dysfunction and Exclusion (TIDE) algorithm was a computational method to model two primary mechanisms of tumor immune escape including the dysfunction of tumor-infiltrating cytotoxic T lymphocytes (CTL) and the rejection of CTL by immunosuppressive factors (17). We applied the algorithm to predict the potential response to immune checkpoint blockade (ICB) therapy (18). Higher TIDE scores indicate worse clinical efficacy of immune checkpoint inhibitors.

Chemotherapeutic response was predicted based on the largest publicly available pharmacogenomics database, the Genomics of Drug Sensitivity in Cancer (GDSC) (https://www.cancerrxgene.org/). We assessed the correlation between DERL3 expression and half-maximal inhibitory concentration (IC50) values of gemcitabine, etoposide and docetaxel, cisplatin, and paclitaxel. The IC50 was estimated by ridge regression. All parameters were set as the default values.

### Single-cell sequencing analysis

The Tumor Immune Single Cell Hub (TISCH, http://tisch.comp-genomics.org) database is a comprehensive web resource enabling interactive single-cell transcriptome visualization of the tumor microenvironment (19). Gene expression data was originated from the GEO database (GSE131907). All downloaded data were uniformly processed with a standardized cell-type annotation and differential expression analysis.

### Gene set enrichment analysis (GSEA)

GSEA was performed using the Java GSEA software v4.1.0 (http://www.broadinstitute.org/gsea). Enrichment map analysis was further applied for GSEA results. A nominal p-value <0.05 and a false discovery rate (FDR) *q* value <0.25 were considered as significant.

### Statistical analysis

The Kaplan–Meier analysis and the log-rank test were applied to identify the associations between DERL3 expression and the prognosis of LUAD patients. The correlation between DERL3 expression and clinicopathological characteristics were statistically analyzed by the χ^2^ test. All data are presented as mean ± standard deviation. All experiments were performed in triplicate. Statistical analysis was conducted using the GraphPad Prism 7.0 software (La Jolla, CA, USA). p < 0.05 was considered as statistically different.

## Results

### The expression profiles and prognostic value of DERL3 in LUAD tissues

**Figure 1:**
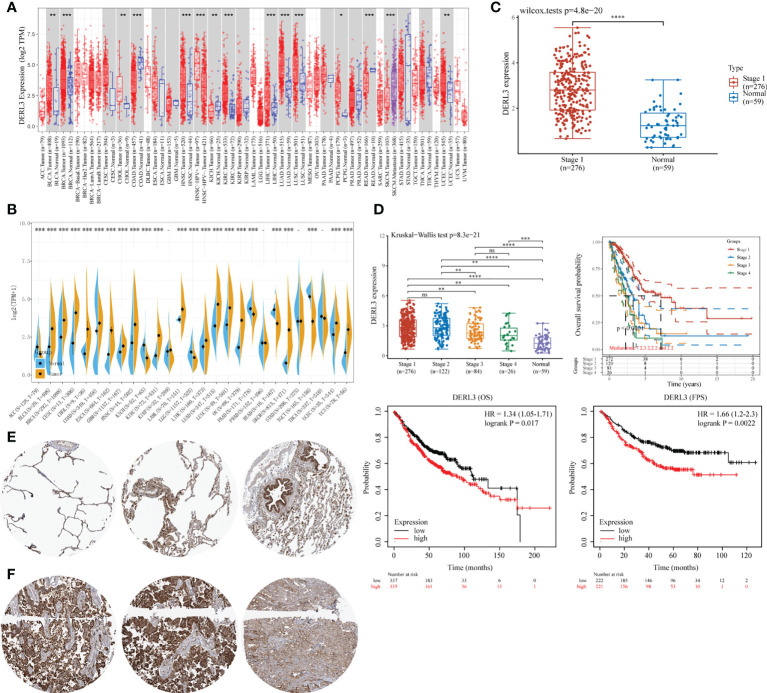
Gene expression and prognostic value of DERL3 in LUAD patients. **(A, B)** Pan-cancer analysis of DERL3 expression across RNA-seq datasets from TCGA. **(C)** Expression of DERL3 in normal pulmonary and stage I LUAD tissues. **(D)** Kaplan–Meier analysis of DERL3 expression with overall survival (OS) and first progression (FP) values in LUAD patients. **(E, F)** Immunohistochemistry of DERL3 expression in normal pulmonary and LUAD tissues based on HPA database. (*p < 0.05, **p < 0.01, ***p < 0.001, ****p < 0.0001, ns, not significant).

DERL3 expression profiles were identified in tumor and normal tissues according to TCGA transcriptomic data. As presented in **Figures 1A, B**, DERL3 expression was elevated in the majority of malignant tissues including LUAD and lung squamous cell carcinoma (LUSC). To further elucidate the expression of DERL3 in different stages of LUAD, RNA-seq dataset of DERL3 was obtained from TCGA. The result revealed that DERL3 was significantly upregulated in stage I LUAD (n = 276) when compared with normal pulmonary tissues (n = 59) (**Figure 1C**). We further identified the protein expression of DERL3 using immunohistochemistry data from the HPA database. The results indicated that DERL3 was medium-stained in normal pulmonary tissues (**Figure 1E**) but strong-stained in LUAD tissues (**Figure 1F**).

The Kaplan–Meier plot was applied to predict the prognostic value of DERL3 in lung cancer patients. It was revealed that the higher level of DERL3 was significantly associated with poorer OS by TCGA LUAD RNA-seq datasets (upper row of **Figure 1D**). Consistently, Kaplan–Meier plotter data also supported that DERL3 was a potential unfavorable prognostic biomarker for OS (HR = 1.34, log-rank p = 0.017) and first progression survival (FPS) (HR = 1.66, log-rank p = 0.0022) in LUAD (bottom row of **Figure 1D**).

### Clinical characterization of DERL3 in LUAD patients

DERL3-related RNA-seq data and clinical phenotypes of LUAD patients were downloaded from the UCSC Xena database. We removed the samples with missing clinical information and integrated the final data. According to the relevant DERL3 expression in tumor tissues, 294 LUAD patients were classified into the DERL3 low expression group (n = 144) and high expression group (n = 150). The correlation between DERL3 expression and clinicopathological characteristics in LUAD patients are shown in **Table 1**. The expression of DERL3 was identified to be correlated with tumor stage (p = 0.032) and T stage (p = 0.024) but not with other clinical characterizations. These results highlighted the clinical significance of DERL3 in LUAD.

**Table 1:** Correlation of DERL3 expression with LUAD clinicopathological characteristics.

Characteristics	No. of patients	DERL3 expression		p value
		Low	High	
Age (year)				
≥60	207	106	101	0.238
<60	87	38	49
Gender				
Male	137	72	65	0.252
Female	157	72	85
Tumor stage				
Stage I-II	228	104	124	0.032*
Stage III-IV	66	40	26
T stage				
T1	102	44	58	0.024*
T2	159	85	74
T3	19	5	14
T4	14	10	4
N stage				
N0	187	86	101	0.319
N1	64	33	31
N2	42	25	17
N3	1	0	1
M stage				
M0	277	132	145	0.066
M1	17	12	5
Primary treatment outcome				
Progressive disease	59	35	24	0.201
Stable disease	27	12	15
Partial remission	3	1	2
Complete remission	205	96	109

*p < 0.05.

### DNA methylation and function enrichment of DERL3 in LUAD

**Figure 2:**
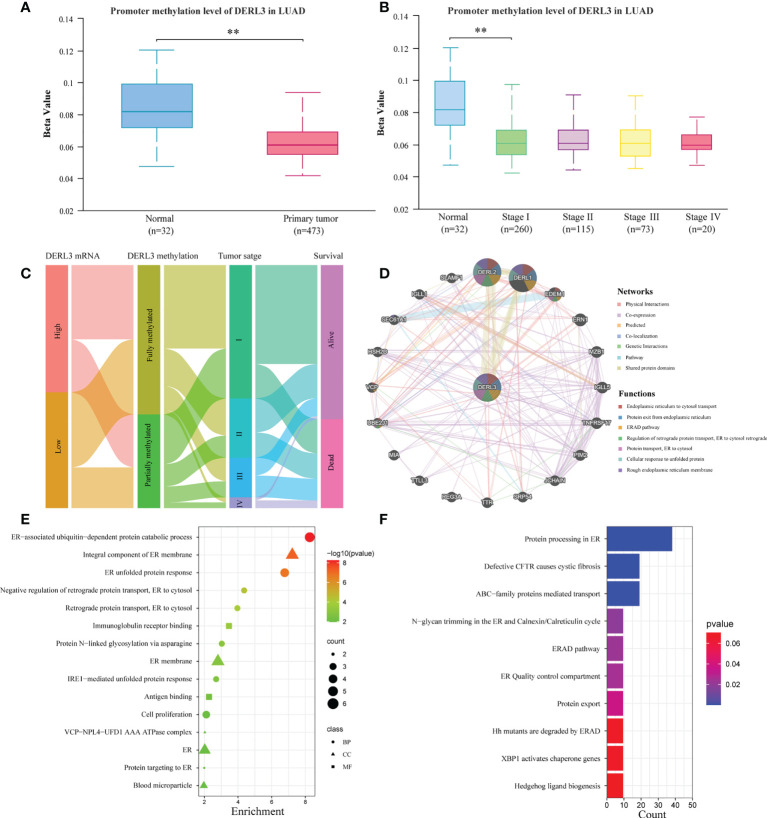
Promoter methylation and functional annotation of DERL3 in LUAD patients. **(A)** The differential methylation of the DERL3 promoter in primary LUAD tumors. **(B)** The correlation between DERL3 promoter methylation and clinical stage of LUAD patients. **(C)** Sankey diagram visualization of relationships between DERL3 mRNA expression, methylation, tumor stage, and survival. **(D)** DERL3 co-expression network constructed by GeneMANIA. **(E, F)** GO and KEGG enrichment analysis of DERL3 co-expression molecules. (**p < 0.01).

DNA methylation is an essential epigenetic regulation mechanism of gene expression. We assessed the promoter methylation level of DERL3 in 32 normal pulmonary tissues and 473 primary LUAD tissues. The results indicated that methylation levels of the DERL3 promoter in LUAD tumors significantly downregulated compared with normal tissues (**Figure 2A**). Moreover, promoter methylation of DERL3 was statistically decreased in clinical stage I LUAD (**Figure 2B**). We further performed the Sankey diagram to identify the association between DERL3 methylation, expression profiles, clinical characteristics, and prognostic signature. As shown in **Figure 2C**, hypermethylation of DERL3 was potentially associated with early stage of lung cancer and expressed an inclination to indicate better survival outcomes.

To further identify the potential protein targets and functional prediction of DERL3 in LUAD, we applied GeneMANIA to generate the protein interaction network. The result indicated that DERL3 was mainly co-expressed with DERL2, DERL1, and EDEM1. Functional prediction revealed that these proteins were all involved in the ERAD process including ER to cytosol transport, protein exit from ER, ERAD pathway, regulation of retrograde protein transport, and cellular response of unfolded protein (**Figure 2D**). We then performed GO and KEGG pathway analysis *via* using the DAVID database. Consistently, GO revealed that DERL3 was correlated with the ER-associated ubiquitin-dependent protein catabolic process, unfolded protein response (UPR), regulation of retrograde protein transport, and IRE1-mediated UPR (**Figure 2E**). Similar to the results obtained from GO annotation, the KEGG pathway indicated that DERL3 was related to the ERAD pathway, ER quality control (ERQC) compartment, and protein export (**Figure 2F**). Moreover, RT-qPCR results presented that the downregulation of DERL3 may significantly alter essential biomarkers of ER stress in LUAD (**Figure S1**).

### DERL3 mediated immune infiltration

**Figure 3:**
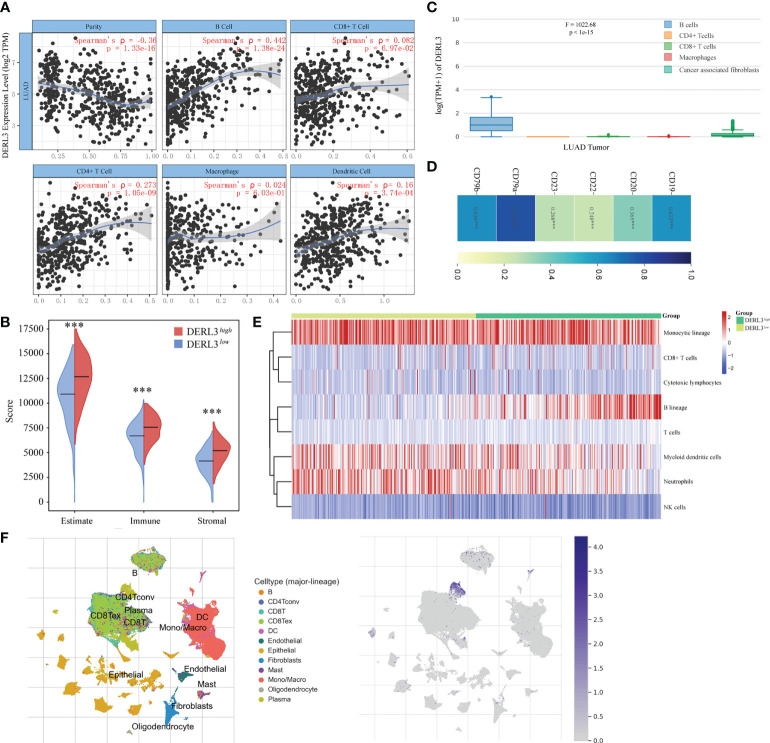
Characteristics of DERL3 in immune cells infiltration. **(A)** Relationship between DERL3 expression and immune infiltration level generated from TIMER. **(B)** Differential expression level of DERL3 with immune scores and stromal scores in LUAD. **(C)** DERL3 expression levels in different immune cells. **(D)** Correlations between cell surface biomarkers of B cells and DERL3 expression. **(E)** Clustering of MCP‐counter scores for the correlation of DERL3 with immune and non-immune stromal cell populations in LUAD according to TCGA databases. **(F)** Distribution of DERL3 gene signature expressed in immune cell clusters based on TISCH databases. (***p < 0.001).

The TIMER database was applied to preliminarily explore whether DERL3 was involved in immune infiltration. The result suggested that DERL3 was positively correlated with immune cells including B cells, CD4+ T cells, CD8+ T cells, macrophages, and dendritic cells, especially in B cells (Spearman’s ρ = 0.442, p = 1.38e-24) (**Figure 3A**). We then evaluated the immune state of DERL3 with the immune score and stromal score, calculated using the ESTIMATE algorithm to estimate infiltrating immune cells and stromal cells in a tumor microenvironment (20). The results demonstrated that the stromal score and immune score significantly differ between the two groups (**Figure 3B**), indicating that DERL3 activation may be linked to immunosuppressive TME. We further explored the correlations between DERL3 and immune cells markers in lung cancer *via* the TIMER (**Table S2**) and GEPIA databases (**Figure 3C**). **Table S2** demonstrated that DERL3 presented significant correlations with B cells, CD8+ T cells, Treg cells (Regulatory T cells), exhausted T cells, TAM (Tumor-associated macrophages), etc. **Figure 3C** illustrated that DERL3 was enriched in B cells.

The above-described results showed that DERL3 may be an immune-related molecule and may appear to be correlated with B cells. We then further investigated the cell surface markers altered with DERL3 activation. In **Figure 3D**, DERL3 was positively correlated with the B cell marker, particularly in CD19 (Spearman’s ρ = 0.623, p < 0.0001), CD79a (Spearman’s ρ = 0.791, p < 0.0001), CD79b (Spearman’s ρ = 0.636, p < 0.0001). This implied that DERL3 was involved in B cell proliferation. We further utilized the MCP counter to analyze the degree of infiltration of immune cells with DERL3 expression. MCP counter is a transcriptome-based computational method that quantifies the abundance of eight major immune and two stromal cell populations from RNAseq tumor bulk (21). As presented in **Figure 3E**, DERL3 was observed to be differentially expressed in B cells. Based on the strong correlation between DERL3 and B cells, T-SNE map was applied to visualize the distribution of DERL3 gene signature expressed in clusters. Results revealed that DERL3 was predominantly enriched in plasma cells of the B cell population (**Figure 3F**).

### Chemotherapy and immunotherapy response

**Figure 4:**
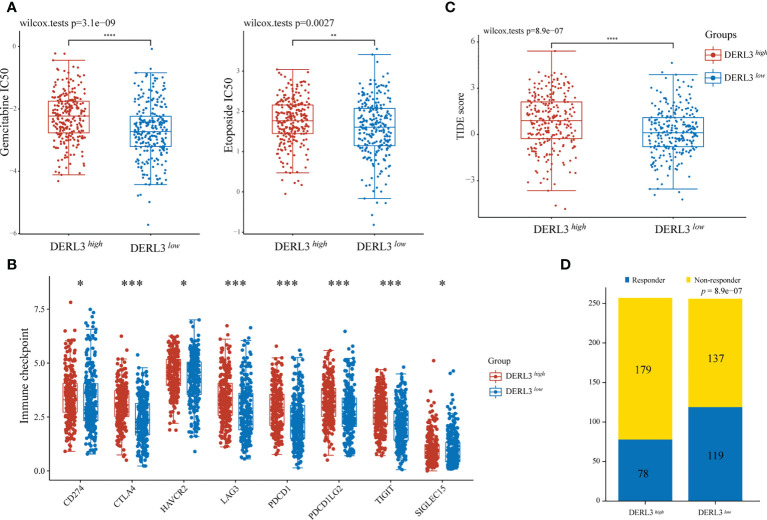
The correlation between DERL3 expression and chemotherapy and immunotherapy. **(A)** The IC50 values of gemcitabine and etoposide toxicity in differentially DERL3 expressed groups. **(B)** Expression of immune checkpoint molecules related to DERL3 differential expression in LUAD patients. **(C)** Prediction of immunotherapy response using the TIDE computational framework. **(D)** Comparison of populations in responders and non-responders to immunotherapy based on TIDE scores. (*p < 0.05, **p < 0.01, ***p < 0.001, ****p < 0.0001).

For medication sensitivity analysis, we assessed the correlation between DERL3 expression and IC50 values of chemotherapeutic agents from GDSC database. The results indicated that the DERL3 low-expression group significantly responded sensitively to gemcitabine, etoposide, and docetaxel, while the tendency was not observed in cisplatin and paclitaxel groups (**Figure 4A**, **Figure S2**). In addition, we evaluated the associations between immune checkpoint molecules and DERL3 expression profiles. It has been demonstrated that the induction of immune checkpoint molecules are inhibitory receptors expressed on immune cells and may trigger immunosuppressive signaling pathways (22, 23). As presented in **Figure 4B**, the expression of DERL3 was elevated in the majority of immune checkpoint molecules, including PD-1, PD-L1, PD-L2, and CTLA-4. It indicated that DERL3 might help the tumor evade from immune surveillance by upregulating immune checkpoint molecules of TME in LUAD. We further applied the TIDE algorithm to predict the response to immunotherapy. Significant differences in response to immunotherapy were observed between the DERL3 high- and DERL3 low-expression groups (**Figure 4C**). Moreover, TIDE results showed higher response rates in the DERL3 low expression group (**Figure 4D**, **Figure S3**, 46.48% vs. 30.35%, p = 8.9e-07). The above-described results revealed that DERL3 might be an indicator of poor therapeutic outcomes.

### Underlying mechanism of DERL3-mediated biological process in LUAD

**Figure 5:**
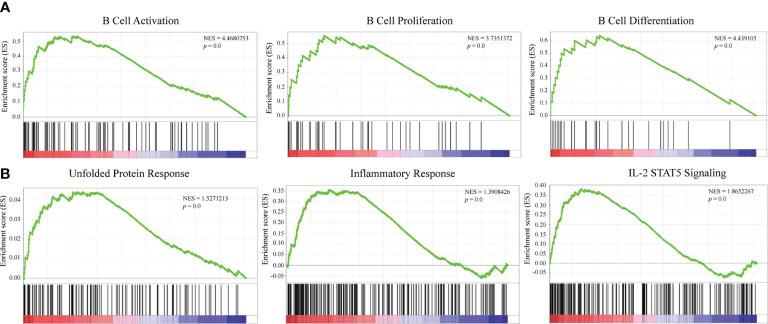
Mechanisms underlying a DERL3-mediated biological process in LUAD. **(A, B)** GSEA was performed using TCGA datasets. The B cell activation, proliferation, and unfolded protein response were identified with the strongest association with DERL3 expression.

GSEA was conducted to explore the potential mechanisms of DERL3-mediated biological process in LUAD. The results indicated that DERL3 was positively correlated with B cell activation (NES = 4.47, p = 0.0), B cell proliferation (NES = 3.74, p = 0.0), and B cell differentiation (NES = 3.74, p = 0.0). Moreover, GSEA also presented that gene signatures of UPR (NES = 1.53, p = 0.0), inflammatory response (NES = 1.39, p = 0.0), and IL-2 STAT5 signaling pathways were enriched when DERL3 was upregulated (**Figures 5A, B** **)**.

### Identification of DERL3 expression in LUAD

**Figure 6:**
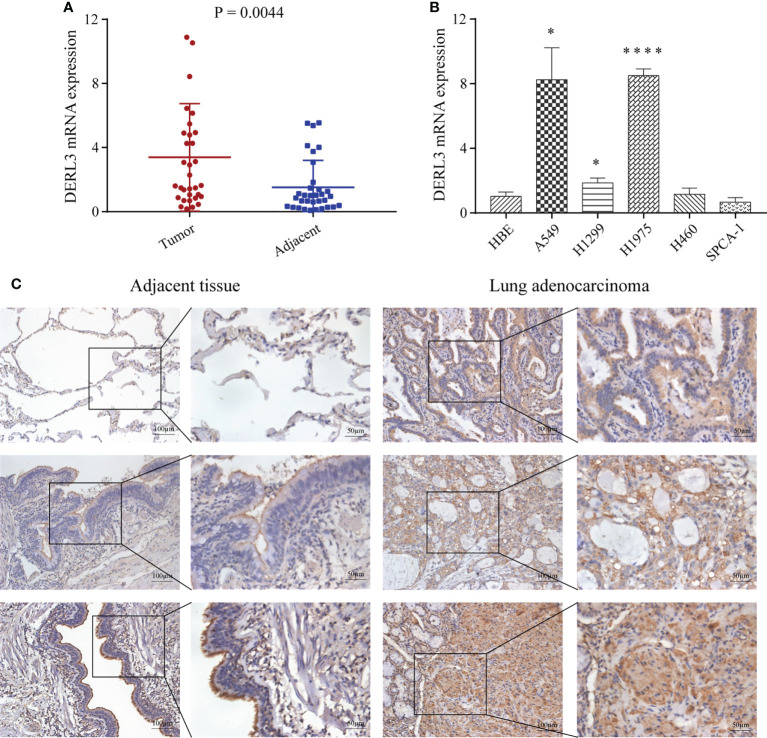
Identification of DERL3 expression in LUAD. **(A)** The mRNA expressions of DERL3 in LUAD tissues compared with adjacent tissues *via* RT-qPCR. **(B)** Detection of DERL3 expression in the normal pulmonary epithelial cell and LUAD cell lines by RT−qPCR. **(C)** Relative protein expression of DERL3 in LUAD tissues compared with adjacent non−tumor tissues with immunohistochemistry. (*p < 0.05, ****p < 0.0001).

We first performed high-throughput sequencing in 10 pairs of stage I LUAD and adjacent non-cancerous lung tissues. The heat map clearly showed a Derlin-related cluster (**Figure S4**), which exhibited that DERL3 was significantly elevated compared with the corresponding adjacent tissue. We then conducted RT-qPCR in 33 paired LUAD and adjacent tissues to preliminarily investigate DERL3 expression. As shown in **Figure 6A**, DERL3 was upregulated in LUAD tissues when compared with adjacent tissues. We further identified DERL3 expression in LUAD cell lines. The results demonstrated DERL3 was upregulated in LUAD cell lines, especially in A549 and H1975 (**Figure 6B**). Additionally, IHC staining also revealed that DERL3 was statistically elevated in LUAD compared with adjacent pulmonary tissues (**Figure 6C**).

### DERL3 modulated LUAD migrative and proliferative capacity

**Figure 7:**
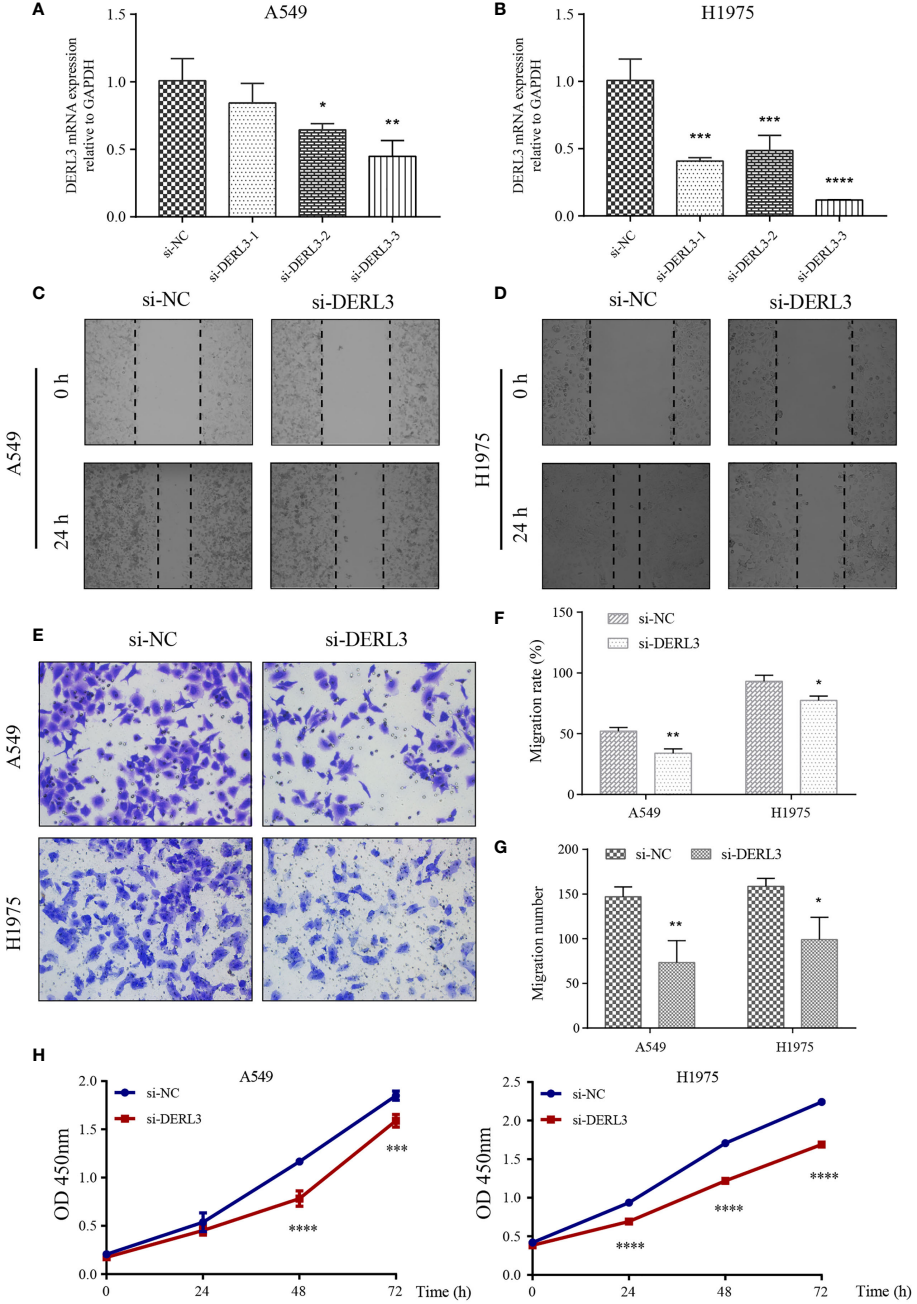
Knockdown of DERL3 inhibited cell migration and proliferation. **(A, B)** Silencing efficiency was identified in A549 and H1975 cells after siRNA transfection. **(C, D)** Scratch assays of A549 and H1975 cells after transfection with si-DERL3. **(E)** Transwell migration assays were performed after transfection with si-DERL3 in A549 and H1975 cells. **(F, G)** Statistical analysis of transwell migration assays and scratch assays were presented. **(H)** CCK-8 assay was performed to evaluate the proliferation of A549 and H1975 cells after silencing DERL3. (*p < 0.05, **p < 0.01, ***p < 0.001, ****p < 0.0001).

We noticed that DERL3 was significantly upregulated in lung cancer cell lines, especially in the A549 and H1975 cell lines. Therefore, A549 and H1975 cells with DERL3 siRNA were constructed using Lipofectamine 3000 transfection. As illustrated in **Figures 7A, B**, the mRNA expression of DERL3 was markedly decreased after si-DERL3-3 transfection in LUAD cells, indicating that the silencing efficacy of si-DERL3-3 was more effective than that of si-DERL3-1 and si-DERL3-2. Migrative ability was evaluated using scratch assays (**Figures 7C, D** **)** and transwell assays (**Figure 7E**). Indeed, the consequences indicated that migratory potentials were reduced by DERL3 knockdown in both A549 and H1975 cells (**Figures 7F, G** **)**. In addition, the proliferation of A549 and H1975 cells were also decreased after DERL3 silencing (**Figure 7H**).

On the other hand, we constructed DERL3 overexpression in SPCA-1 cells. The efficacy was confirmed by the elevated level of DERL3 mRNA expression (**Figure S5A**). The CCK-8 assay demonstrated that overexpression of DERL3 enhanced the proliferation of SPCA-1 cells (**Figure S5B**). Additionally, scratch and transwell assays presented that the migrative ability of SPCA-1 cells was significantly enhanced when compared with the control cells (**Figures S5C-F**). Collectively, the above consequence supported that DERL3 exerted a critical role in LUAD occurrence and progression.

## Discussion

Chronic and persistent ER stress has emerged as an essential pathophysiological paradigm underlying lung cancer (24, 25). DERL3, as an important element in ER homeostasis, was rarely investigated in previous molecular studies (26). Herein, we comprehensively explored gene expression, molecular function, and immune infiltration of DERL3 in LUAD. Our results identified that DERL3 was upregulated in lung cancer and high expression levels of DERL3 predicted an adverse prognosis of LUAD patients. Functional analysis revealed that DERL3 was associated with the ERAD process and involved in immune infiltration. Therefore, we speculated that DERL3 might serve as an oncogenic molecule in immune suppressive TME by inducing the ERAD process.

Researches have demonstrated that the Derlin protein superfamily was involved in the ERAD process of cancer cells (27, 28). Tumor cells exposed to nutrient-deprived and inflammatory TME are more prone to activating ER stress (29, 30). Unchecked accumulation of aberrant proteins generates constant ER stress and underlies the most pressing maladies, including aging, cancer, and neurodegenerative diseases (31–33). To offset the catastrophic effect of unwanted proteins, ER is equipped with protein quality control systems for surveillance, including ERAD (34). DERL3 has emerged as a potential candidate for retro-translocating ERAD substrates tagged with ubiquitin out of the ER (35). Our results indicated that DERL3 was elevated in LUAD and associated with inflammatory TME. Therefore, we supposed that DERL3 differential expression may be attributed to the chronic inflammation of TME, inducing the activation of ER tress. Co-expressed molecules of DERL3 were primarily involved in the ERAD process; GO and KEGG annotation further implied that DERL3 was mainly enriched in ER-associated protein ubiquitination and ERAD. GSEA presented consistent consequences that DERL3 was related with UPR, an essential preventative system of the ER-induced quality control pathway. The above consequences support the hypothesis that DERL3 located in ER membrane can aid in the proteasomal degradation of proteins.

Infiltrating immune cells are integral components of TME (36). A previous study has reported that cancer-derived factors in TME might trigger ER stress in innate immune cells to blunt anti-tumor immunity (37), which suggested an interplay between ER stress and immune cells. Interestingly, our results indicated that DERL3 was related to immune cell infiltration including B cells, CD4+ T cells, dendritic cells, and macrophages, particularly enriched in B cells. In addition, the efficacy of immune checkpoint blockade and chemotherapy was negatively related to the expression of DERL3. Consistently, Francesca et al. ascertained that DERL3 was elevated in plasma cells *via* RNA-seq identification (38). Moreover. Kriss et al. reported Derlin family members, DERL1 and DERL2, were transcriptionally upregulated in Eμ-TCL1-expressed murine B cells of mouse chronic lymphocytic leukemia (39), while Dougan et al. demonstrated that DERL2 absent in B lymphocytes may not alter cell development and antibody secretion (40). The potential association between DERL3 and B cell has not ever been reported; the exact mechanism of why DERL3 enriched in B cells remains unknown. Herein, we speculated that plasma cells need to expand the ER and Golgi networks to sustain antibody secretory capacity, whereas excessive production of immunoglobulins and ER remodeling may cause ER stress and trigger the UPR (41, 42), which may contribute to the elevated expression of the ER stress central regulatory molecule, including DERL3. However, this assumption still needs to be validated *in vivo* and *in vitro*.

The present study remains several potential limitations listed as follows: Firstly, our results and conclusions lack of experimental validation and prospectively clinical cohort. Secondly, the heterogeneity of analytical variations, database application and sample heterogeneity may unintentionally impact the results. Our research was mainly summarized based on the public datasets, further studies based on experimental samples are required.

## Conclusion

In conclusion, we demonstrated that high expression of DERL3 predicted an adverse outcome of LUAD patients. Moreover, functional enrichment analysis indicated that differentially expressed DERL3 was involved in ER stress, particularly in the ERAD process. Besides, DERL3 was proven to be associated with immune infiltration in suppressive TME, and overexpression of DERL3 may be an indicative predictor of immunotherapy resistance. These findings indicate a promising beginning for the discovery of potential prognostic predictors and immunotherapeutic strategies for LUAD.

## Data availability statement

The datasets presented in this study can be found in online repositories. The name of the repository and accession number can be found below: National Center for Biotechnology Information (NCBI) BioSample, https://www.ncbi.nlm.nih.gov/biosample/, SAMN19324451.

## Author contributions

YZ and YX conceived and supervised the project. LL and GL performed the experiments, analyzed the data, and wrote the manuscript. HL assisted with experiments. LC and XC assisted with the computational analysis. All authors read and approved the final manuscript.

## Funding

This work was supported by the Fujian Provincial Health Fund for Young and Middle-aged People (2019-ZQNB-7) and Startup Fund for scientific research, Fujian Medical University (Grant number: 2021QH2044).

## Conflict of interest

The authors declare that the research was conducted in the absence of any commercial or financial relationships that could be construed as a potential conflict of interest.

## Publisher’s note

All claims expressed in this article are solely those of the authors and do not necessarily represent those of their affiliated organizations, or those of the publisher, the editors and the reviewers. Any product that may be evaluated in this article, or claim that may be made by its manufacturer, is not guaranteed or endorsed by the publisher.
