# Supplementary material for: Integrated profiling of endoplasmic reticulum stress-related DERL3 in the prognostic and immune features of lung adenocarcinoma
Source: Front Immunol. 2022 Oct 7;13:906420. doi: 10.3389/fimmu.2022.906420 (PMC9585215; doi:10.3389/fimmu.2022.906420)
Supplement: Supplementary file 1 [file DataSheet_1.pdf]

![frontiers]

Supplementary Material

Figure S1. Essential biomarkers of ER stress altered with DERL3 expression (**P* < 0.05, ****P* < 0.001, *****P* < 0.0001).

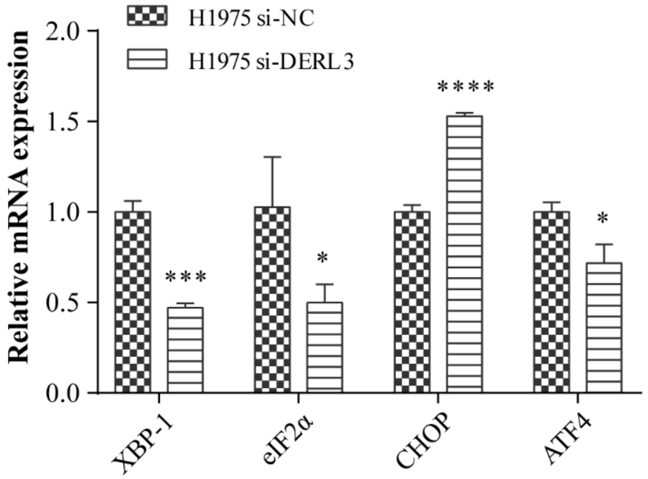

Figure S2. IC50 values of docetaxel, cisplatin and paclitaxel on LUAD tissues (**P* < 0.05, ***P* < 0.01).

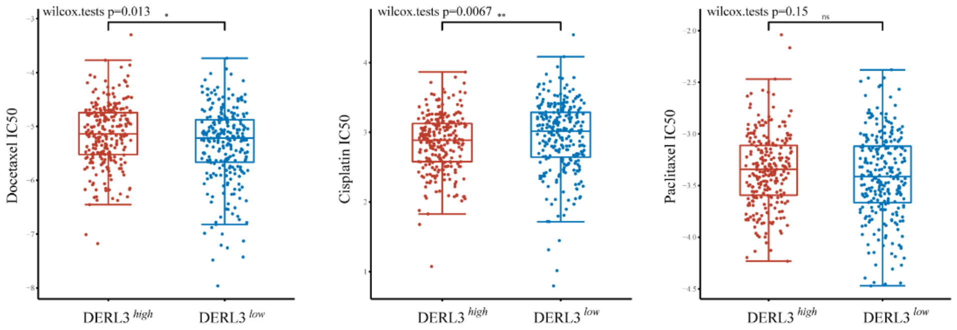

Figure S3. TIDE computational framework of responders and non-responders.

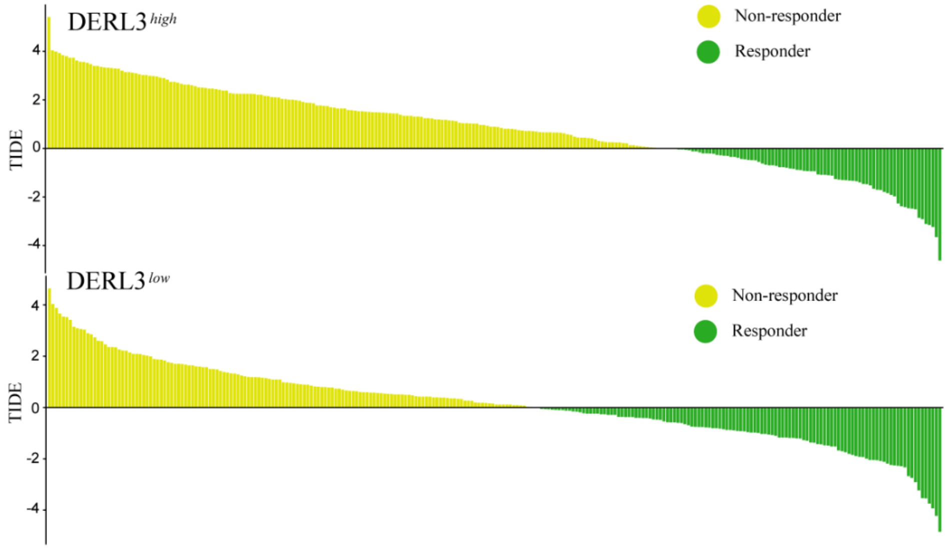

Figure S4. Heat maps of Derlin protein profiles identified in LUAD and adjacent non-cancerous tissues.

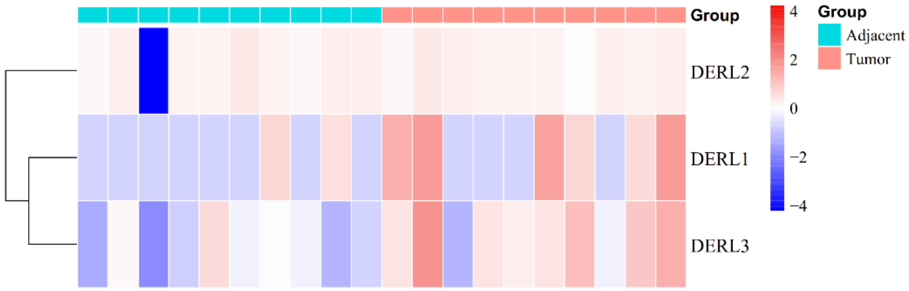

Figure S5. Overexpression of DERL3 enhanced SPCA-1 migration and proliferation. (a) Overexpression efficiency was identified in SPCA-1 cells after DERL3-flag plasmid transfection. (b) CCK-8 assay was performed to evaluate the proliferation of SPCA-1 cells after DERL3 overexpression. (c) Scratch assays of SPCA-1 cells after transfection with DERL3-flag plasmid. (d) Transwell migration assays were performed in SPCA-1 cells. (e-f) Statistical analysis of scratch assay and transwell migration assay. (*$P < 0.05$, **$P < 0.01$, ****$P < 0.0001$).

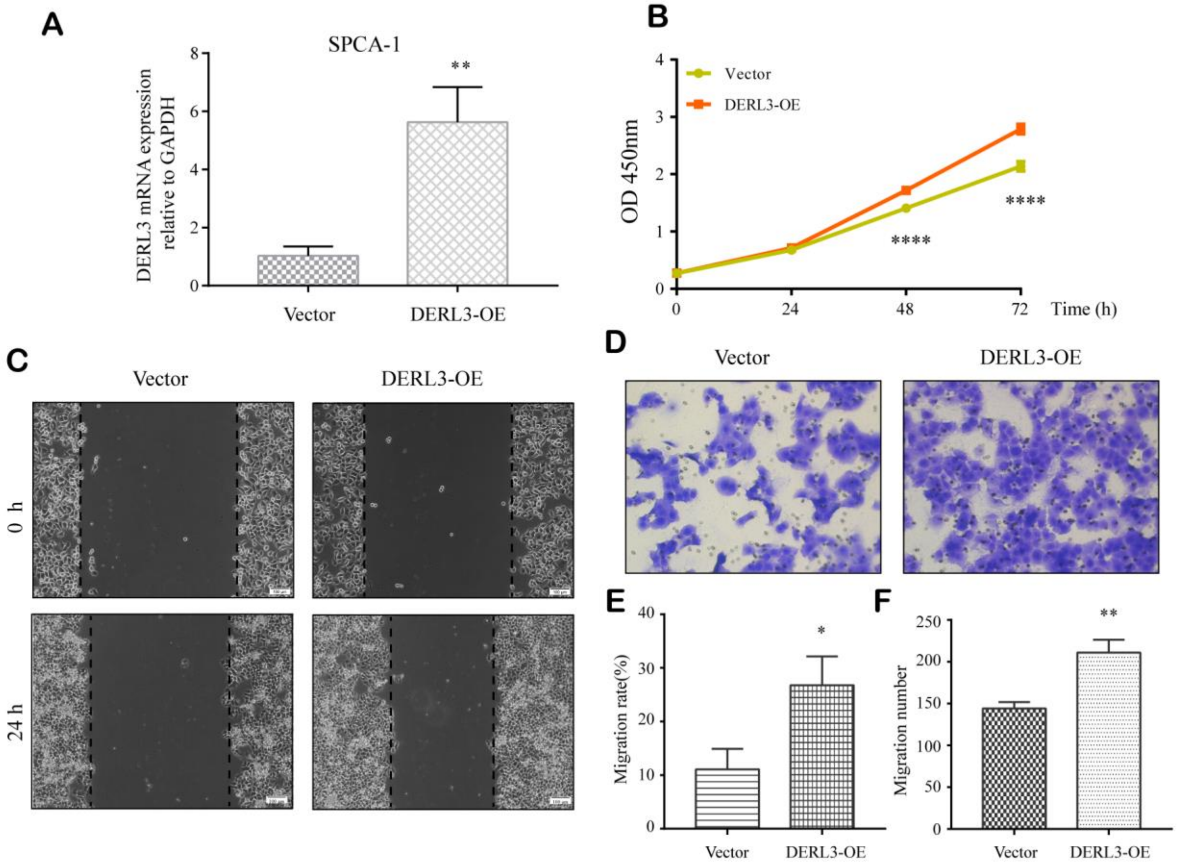

Table S1. Sequence information

Gene	Forward (5’-3’)	Reverse (5’-3’)
Si-DERL3-1	CCCCUUUCAACUCUACUUCAATT	UUGAAGUAGAGUUGAAAGGGGTT
Si-DERL3-2	GAUUCAGCUUCUUCUUCAATT	UUGAAGAAGAAGCUGAAUCTT
Si-DERL3-3	CUAGAUCUUCAGUGCUGUAUGTT	CAUACAGCACUGAAGAUCUAGTT
Si-NC	UUCUCCGAACGUGUCACGUTT	ACGUGACACGUUCGGAGAATT
DERL3	TCTGGAAGGAGAACAGGCTGAGG	AGGTGGTATGTGAACAAGGTTGGC
XBP-1	CCTGGTTGCTGAAGAGGAGG	CCATGGGGAGATGTTCTGGAG
eIF2α	GCTGTGACACCGTAACTCTT	CCACAGGTTTCTCAGGTAAG
CHOP	AGGCGACCAAACTAAAGAAGG	TGCTGAGTTGAGCGTTATACTG

ATF4	ATGACCGAAATGAGCTTCCTG	GCTGGAGAACCCATGAGGT
GAPDH	CATGTTCGTCATGGGTGTGAACCA	AGTGATGGCATGGACTGTGGTCAT

Table S2. Correlations between DERL3 and gene markers of infiltrating immune cells.

Cell	Gene marker	Without adjustment		Purity adjustment	
		Spearman's ρ	*P*	Spearman's ρ	*P*
B cell	CD19	0.679	5.49E-71 ****	0.623	2.00E-54 ****
	MS4A1	0.464	6.60E-29 ****	0.365	5.13E-17 ****
	CD22	0.364	1.51E-17 ****	0.248	2.3E-08 ****
	CD23	0.362	2.15E-17 ****	0.268	1.49E-09 ****
	CD79A	0.816	1.94E-124 ****	0.791	7.88E-107 ****
	CD79B	0.686	7.43E-73 ****	0.636	3.49E-57 ****
CD8+ T cell	CD8A	0.339	2.35E-15 ****	0.216	1.35E-06 ****
	CD8B	0.335	5.95E-15 ****	0.241	6.19E-08 ****
Th1	IL-2	0.208	1.87E-06 ****	0.149	0.0009 ***
Th2	IL-4	0.008	0.859	0.014	0.763
	IL-5	0.061	0.163	0.036	0.419
Treg	FOXP3	0.451	3.40E-27 ****	0.349	1.46E-15 ****
	CCR8	0.377	7.67E-19 ****	0.281	2.04E-10 ****
	IL-7R	0.356	7.86E-17 ****	0.238	9.38E-08 ****
T cell exhausted	LAG3	0.384	1.68E-19 ****	0.281	2.05E-10 ****
	CTLA4	0.421	1.72E-23 ****	0.308	2.86E-12 ****

DC	CD1C	0.075	0.089	-0.004	0.927
Macrophage	CD68	0.165	0.0002 ***	0.051	0.258
M1	NOS2	0.128	0.004 **	0.052	0.253
	ROS1	-0.018	0.681	-0.077	0.086
	IL-12B	0.144	0.001 **	0.046	0.312
	HLA-DR	0.227	1.81E-07 ****	0.116	0.010 **
M2	ARG1	0.055	0.213	0.0498	0.270
	MRC1	0.066	0.135	-0.031	0.490
	CD68	0.165	0.0002 ***	0.051	0.258
	CD163	0.163	0.0002 ***	0.046	0.310
TAM	HLA-G	0.147	0.0008 ***	0.068	0.130
	CD80	0.215	7.97E-07 ****	0.083	0.067
	CD86	0.248	1.25E-08 ****	0.115	0.010 *
MDSC	CD33	0.134	0.002 **	0.011	0.804
M-MDSC	CD14	0.292	1.51E-11 ****	0.183	4.20E-05 ****

Treg, Regulatory T cells; DC, Dendritic cells; TAM, Tumor associated macrophage; MDSC, Myeloid-derived suppressor cell; M-MDSC, Monocytic myeloid-derived suppressor cell; *$P < 0.05$; **$P < 0.01$; ***$P < 0.001$; ****$P < 0.0001$.
